# Supplementary material for: Variations induced by the use of unstable surface do not facilitate motor adaptation to a throwing skill
Source: PeerJ. 2023 Jan 13;11:e14434. doi: 10.7717/peerj.14434 (PMC9841905; doi:10.7717/peerj.14434)
Supplement: Supplemental Information 2 — Student t tests for repeated measures comparing between test conditions (throwing on the floor vs. throwing on the BOSU) o pre and post intervention tests. 1 t, p, and effect size values refer to pair comparisons between test conditions (throwing on the floor vs. throwing on the BOSU). 2 t, p, and effect size values refer to pair comparisons between pre and post intervention tests. Hedges’ g index was used to estimate the effect size of each pair comparison using the standard deviation of the change between repeated measure conditions. [file peerj-11-14434-s002.docx]

| **Supplementary material 1.** Mean radial error in pre-test and post-test according to the throwing surface and the practice group. Units in cm | | | |
| --- | --- | --- | --- |
|  | **ON-FLOOR TRAINING GROUP (n=9)** | | |
|  | **Pretest** | **Posttest** | **t (p)**  **Effect size^2^** |
| **Throwing on the FLOOR** | 8.40 ± 3.47 | 5.61 ± 1.64 | 3.362 (0.010)  1.1067 |
| **Throwing on the BOSU** | 11.21 ± 5.43 | 7.07 ± 2.97 | 4.523 (0.002)  1.436 |
| **t (p)** | -2.624 (0.30) | -2,920 (0.019) |  |
| **Effect size^1^** | -0.833 | -0.927 |  |
|  | **ON-BOSU TRAINING GROUP (n=12)** | | |
|  | **Pretest** | **Posttest** | **t (p)**  **Effect size^2^** |
| **Throwing on the FLOOR** | 8.18 ± 3.83 | 6.15 ± 2.58 | 2.488 (0.030)  0.693 |
| **Throwing on the BOSU** | 9.32 ± 3.93 | 6.57 ± 2.69 | 2.867 (0.014)  0.799 |
| **t (p)** | -2.655 (0.22) | -1,152 (0.274) |  |
| **Effect size^1^** | -0.740 | -0.321 |  |
| *Student t tests for repeated measures comparing between test conditions (throwing on the floor vs. throwing on the BOSU) o pre and post intervention tests.*  *^1^ t, p, and effect size values refer to pair comparisons between test conditions (throwing on the floor vs. throwing on the BOSU).*  *^2^ t, p, and effect size values refer to pair comparisons between pre and post intervention tests.*  *Hedges’ g index was used to estimate the effect size of each pair comparison using the standard deviation of the change between repeated measure conditions.* | | | |
